# Detection of human cytomegalovirus in patients with epithelial ovarian cancer and its impacts on survival

**DOI:** 10.1186/s13027-020-00289-5

**Published:** 2020-04-15

**Authors:** Min Yin, Aiping Chen, Fei Zhao, Xuechao Ji, Chuan Li, Guangning Wang

**Affiliations:** 1 Medical College of Qingdao University, Qingdao, Shandong Province China; 2 Department of Gynecology, The Affiliated Hospital of Qingdao University, Qingdao, Shandong Province China

**Keywords:** Epithelial ovarian cancer, Human cytomegalovirus, Viral carcinogenesis, Survival

## Abstract

**Background:**

The cause of epithelial ovarian cancer (EOC) is not elucidated. Viral infection may induce chronic inflammatory infection and play a role in the pathogenesis of cancers. Some viruses are considered to be oncomodulatory, modulating cellular pathways such as cell proliferation, tumor progression, vascular disease development, and immune evasion. Human cytomegalovirus (HCMV) has been detected in several types of cancers including ovarian cancer. However, the role of HCMV in ovarian carcinogenesis remains controversial.

**Objective:**

To investigate the potential role of HCMV infection in EOC, we evaluated the prevalence of HCMV proteins in EOC tissue and its impacts on patients’ survival.

**Methods:**

Formalin-fixed paraffin-embedded tissues from 66 patients with EOC and 30 patients with benign ovarian cystadenoma were studied. Specimens were analyzed for expression of HCMV immediate early protein (IE) and HCMV tegument protein (pp65) by immunohistochemistry.

**Results:**

HCMV-IE protein expression was detected in 82% of EOC and 36% of benign tumors; pp65 was detected in 97% of EOC and 63% of benign tumors. Extensive HCMV-IE protein expression was associated with higher stage of EOC. Reactivation of latent HCMV within the tumor at interval debulking surgery may be induced by neoadjuvant chemotherapy before surgery. Extensive HCMV-IE expression was associated with shorter median overall survival than focal or negative expression (39 versus 41 months, *P* = 0.03). Multivariate analysis indicated that HCMV-IE expression was an independent prognostic factor for overall survival (*P* = 0.034).

**Conclusions:**

This study demonstrate a high prevalence of HCMV proteins in tissue sections from patients with EOC. HCMV infection can be potential risk factor for EOC development. Extensive HCMV-IE expression indicated a poor prognosis. The relationship between HCMV and clinical outcomes highlight the need for further researches on the oncomodulatory role of HCMV in ovarian cancer.

## Introduction

Ovarian cancer is a major cause of cancer deaths in women. Patients are often diagnosed with advanced-stage due to the lack of effective screening methods and the non-specific symptoms. Ovarian cancer is a highly fatal disease, with a global 5-year survival rate of 30–40% in women at advanced stages of diagnosis [1]. However, our understanding of the exact cause of ovarian cancer is limited. During recent years, serous tubal intraepithelial carcinomas (STIC) have been shown to be precursor lesions of serous EOC [2]. Anatomically, the female peritoneal cavity and internal genitalia are accessible to outside pathogens through the genital tract. Furthermore, the fallopian tubes are easily affected by pelvic inflammatory disease (PID), it is therefore highly hypothesized that microbial infection may contribute to ovarian cancer [3].

HCMV is a member of the β-herpesviruses family, which can establish life-long latency. If the patient’s immunological status is impaired, the viral replication cycle will be reactivated [4]. During active infection, HCMV expresses several proteins, some are essential for its replication and a large amount may interfere with the cellular and immunological functions, enabling the virus to coexist with its host [5]. Recently, several studies provide evidence that HCMV proteins and nucleic acid has been detected in tissue from several malignancies, including cervical, breast, colorectal, as well as glioblastoma and neuroblastoma [6–10]. Shanmughapriya et al. first found HCMV-glycoprotein DNA by polymerase chain reaction analysis in 50% of tumor tissue specimens from ovarian cancer patients [11]. Carlson et al. reported that HCMV proteins and nucleic acids are frequently detected at different levels in high grade serous ovarian carcinoma, and shorter median overall survival was shown in patients with positive HCMV IE and pp65 [12]. Lately, Paradowska et al. analyzed the prevalence of human papillomavirus (HPV) and HCMV in EOC tissue and fallopian tube specimens obtained at tumor resection [13]. The presence of HCMV and HPV DNA was detected in 70 and 74% cancerous ovarian tissues, respectively, and was significantly higher in EOC than in benign tumor cases. HCMV or HPV infection was observed also in the fallopian tube samples. Two thirds of EOC patients demonstrated coinfection with HCMV and HPV in the pathological samples, suggesting that the infections of HCMV and HPV can be potential risks for EOC development. However, Ingerslev et al. examined the prevalence of Epstein-Barr Virus (EBV) DNA and HCMV DNA in EOC tissue samples, HCMV DNA was detected in only one case sample (0.5%), showing no association between HCMV and EOC [14].

To elucidate the potential role of HCMV in ovarian cancer and possible impact of HCMV infection on the clinical outcomes, we investigated the prevalence of HCMV proteins in EOC tissue and compared findings to those obtained in benign ovarian cystadenoma.

## Materials and methods

### Clinical samples

Between January and December 2015, 66 patients with EOC and 30 patients with benign ovarian cystadenoma were enrolled in the study. All patients underwent surgery and received treatments at the Gynecology Department in The Affiliated Hospital of Qingdao University. Thirty-four patients had primary debulking surgery, and 32 had interval debulking surgery after NACT. All patients received conventional adjuvant chemotherapy after the surgery. Carboplatin AUC5 and paclitaxel 175 mg/m^2^ intravenously every third week in total 6 to 8 cycles is recommended according to the guidelines. The clinical follow-up continued to December 2019. Either re-examination in the outpatient or phone interview was used to determine the patients’ condition during the follow-up. Overall survival (OS) was defined as the time interval from the date of diagnosis with clinical pathology to the date of EOC-related death or the last follow-up. The study was approved by the medical ethics committee of The Affiliated Hospital of Qingdao University. Since all specimens were collected anonymously, the Medical Ethics Committee exempted patients from the need for informed consent.

### Immunohistochemical analyses of paraffin sections

Formalin-fixed paraffin-embedded surgical specimens were obtained from the pathology tissue archives of the hospital. Four micrometer paraffin sections were deparaffinized in the xylene, and hydrated them in graded alcohols. For antigen retrieval, tissue sections were performed by treatment with pepsin (BioSite) at 37 °C for 15 min, at pH 7.6 and overnight incubation at 37 °C. Endogenous nonspecific binding of antibodies was blocked with 3% H_2_O_2_ (Bioss, China), avidin/biotin blocking reagents (Bioss, China), FC receptor blocker (Bioss, China). Monoclonal antibodies against HCMV IE protein (Millipore, USA) and HCMV tegument protein pp65 (Bioss, China) were used for the detection of different HCMV proteins. The extent of HCMV infection was scored as follows: negative (0% positive cells), focal (< 50% positive cells), or extensive (≥50% positive cells), estimating from the number of cells expressing HCMV proteins, according to the score criteria from Radestad et al. [15]. All staining results were independently reviewed by two pathologists.

### Statistical analyses

Immunohistochemical data were determined in EOC and benign ovarian cystadenoma samples. All statistical analyses were performed using SPSS version 19.0 (SPSS Inc., Chicago, IL, USA). Chi-square tests were applied to analyse categorical data. OS was presented as Kaplan–Meier survival curves. The statistical significance of survival data was determined by log-rank test. Multivariate Cox proportional hazards regression model was used to assess the independent predictive factors. *P* < 0.05 was considered significant.

## Results

### Patients characteristics

The mean age of the 66 patients with EOC was 57 years old, ranged from 45 to 71 years. At study closure, 25 out of 66(38%) patients were alive. Thirty patients with benign ovarian cystadenoma served as controls. The mean age of the controls was 43 years old, ranged from 16 to 62 years. The detailed information was shown in Table 1.

**Table 1:** Patients characteristics

Patients characteristics	
**Epithelial ovarian cancer(*n*** **= 66)**	**n(%)**
Age	
< 50y	8
50-60y	24
60-70y	32
≥ 70y	2
Initial CA125	
< 35 U/ml	4
≥ 35 U/ml	62
Pathological type	
Serous adenocarcinoma	54
Mucinous adenocarcinoma	2
Endometrioid adenocarcinoma	2
Clear cell carcinoma	8
Stage	
I,II	8
III,IV	58
Neoadjuvant chemotherapy before surgery	32
**Benign ovarian cystadenoma (*n*** **= 30)**	
Age	
10-30y	5
30-50y	6
> 50y	19
Initial CA125	
< 35 U/ml	26
≥ 35 U/ml	4
Pathological type	
Serous cystadenoma	13
Mucinous cycstadenoma	17

### Immunohistochemistry for HCMV

**Fig. 1:**
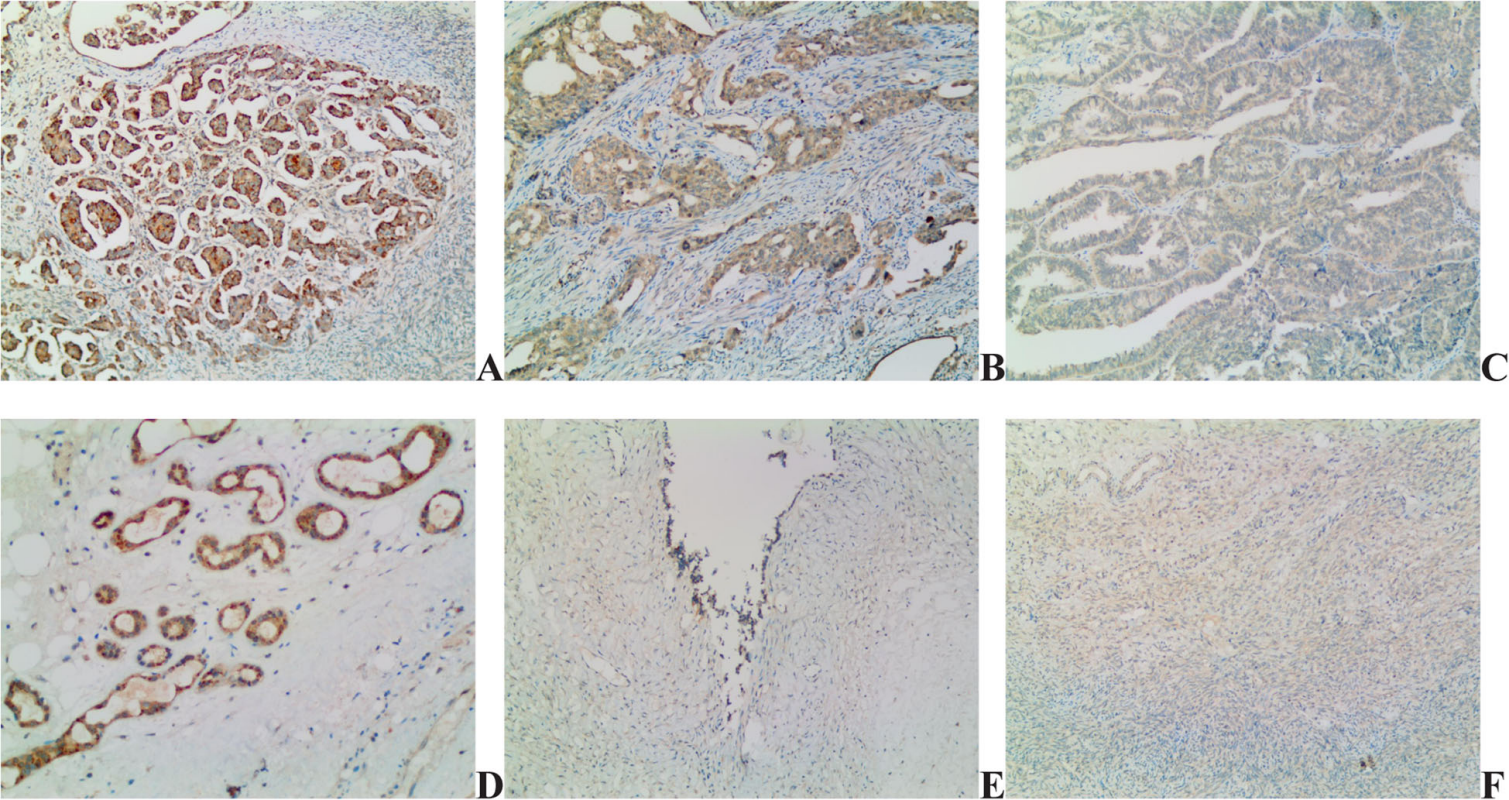
Detection of HCMV-IE in EOC tissue and benign ovarian cytstadenoma. HCMV IE extensive (**a**), focal (**b**) and negative (**c**) expression in EOC. HCMV IE extensive (**d**), focal (**e**) and negative (**f**) expression in benign ovarian cystadenoma sections (10 × 10)

**Fig. 2:**
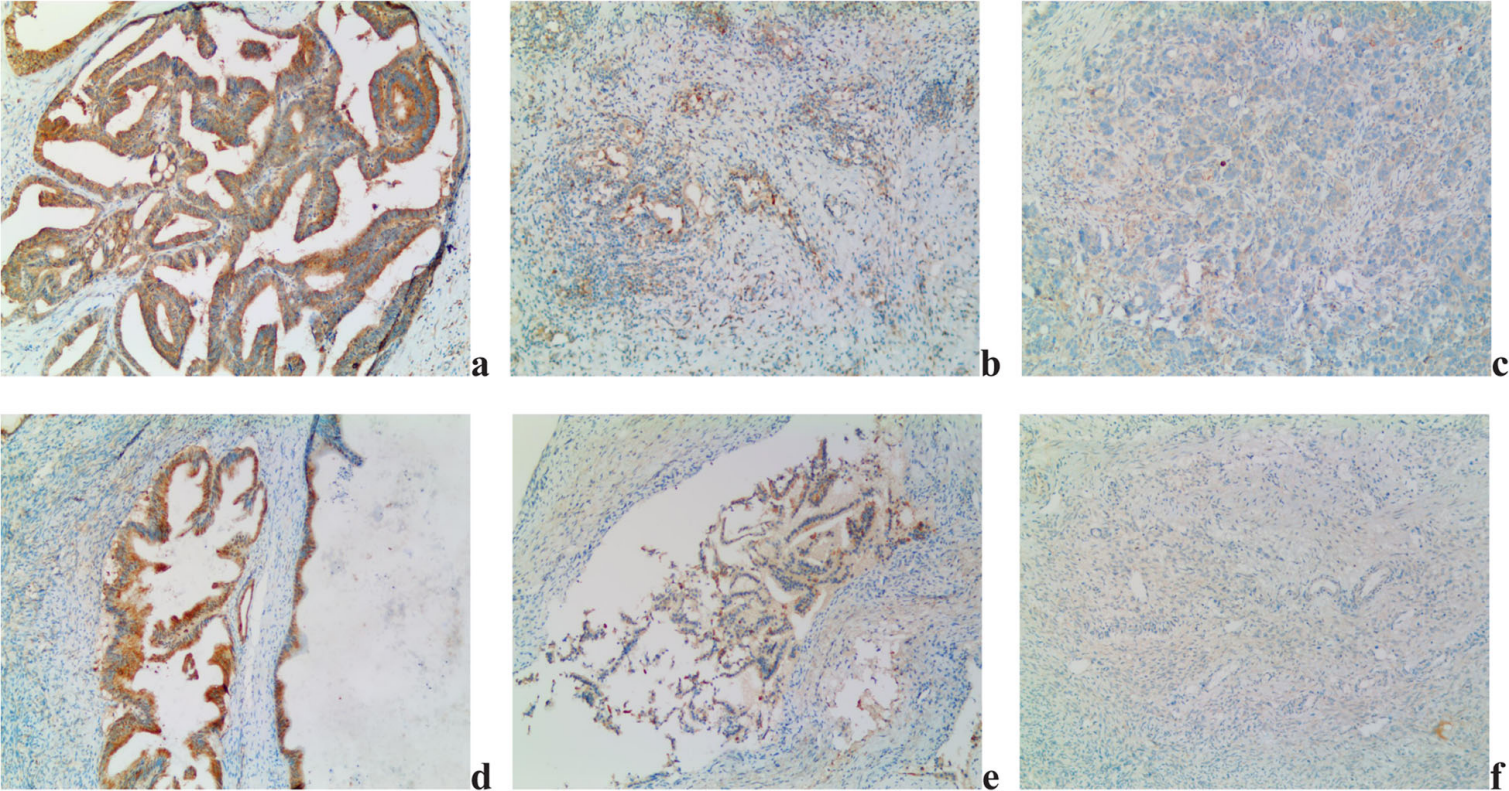
Detection of HCMV-pp65 in EOC tissue sections and benign ovarian cytstadenoma. HCMV pp65 extensive (**a**), focal (**b**) and negative (**c**) expression in EOC. HCMV pp65 extensive (**d**), focal (**e**) and negative (**f**) expression in benign ovarian cystadenoma sections (10 × 10)

HCMV-IE protein was detected from 82% of EOC patients and 36% of patients with benign cystadenoma (Table 2). HCMV-IE protein expression was extensive in 61%, focal in 21% and negative in 18% of EOC tissues. Moreover, expression was extensive in 23%, focal in 13% and negative in 64% of benign ovarian cystadenoma (Fig. 1). HCMV-pp65 protein was detected in tumor specimens from 97% of EOC patients and 63% of those with benign cystadenoma (Table 2). The expression was extensive in 76%, focal in 21% and negative in 3% in epithelial ovarian cancer tissue. Furthermore, expression was extensive in 33%, focal in 30% and negative in 37% in benign ovarian cystadenoma (Fig. 2).

**Table 2:** Expression of HCMV-IE and pp65 in EOC and Benign Ovarian Cystadenoma

Type of tumor	HCMV IE			HCMV PP65		
	Extensive, n(%)	Focal, n(%)	Negative, n(%)	Extensive, n(%)	Focal, n(%)	Negative, n(%)
EOC	40/66(61)	14/66(21)	12/66(18)	50/66(76)	14/66(21)	2/66(3)
Ovarian cystadenoma	7/30(23)	4/30(13)	19/30(64)	10/30(33)	9/30(30)	11/30(37)

### Association between high HCMV expression proteins and advanced disease

Next we analyzed the effects of HCMV on the EOC stage. HCMV-IE expression was extensive in 25% of Stage I-II tumors, 66% of Stage III-IV tumors; HCMV-pp65 expression was extensive in 38 and 64% of stage I-II and stage III-IV tumors, respectively. We observed that advanced tumor stage was correlated with extensive expression of HCMV-IE (*P* = 0.0279) (Table 3).

**Table 3:** Expression of HCMV-IE and pp65 in EOC tissues of different stages

	Extensive, n(%)	Focal/negative, n(%)	Chi-square	P
HCMV-IE			4.834	0.0279
I,II	2(25)	6(75)		
III,IV	38(66)	20(34)		
HCMV-pp65			2.036	0.1536
I,II	3(38)	5(62)		
III,IV	37(64)	21(36)		

### Reactivation of latent HCMV and NACT

HCMV-IE expression was extensive in 75% of cancer tissue with NACT before surgery, 47% of cancer tissue without NACT; HCMV-pp65 expression was extensive in 69, and 53%, respectively. This observation indicates that reactivation of latent HCMV within the tumor at interval debulking surgery (IDS) may be induced with NACT as HCMV-IE viral proteins could be significantly extensive expressed in tumor tissue sections with NACT before surgery(*P* = 0.0279) (Table 4).

**Table 4:** Expression of HCMV-IE and pp65 in EOC tissues with/without NACT

	Extensive, n(%)	Focal/negative, n(%)	Chi-square	P
HCMV-IE			5.390	0.0202
NACT before surgery	24(75)	8(25)		
No NACT before surgery	16(47)	18(53)		
HCMV-pp65			1.726	0.1890
NACT before surgery	22(69)	10(31)		
No NACT before surgery	18(53)	16(47)		

### Survival rate among EOC patients with extensive HCMV-IE expression

**Fig. 3:**
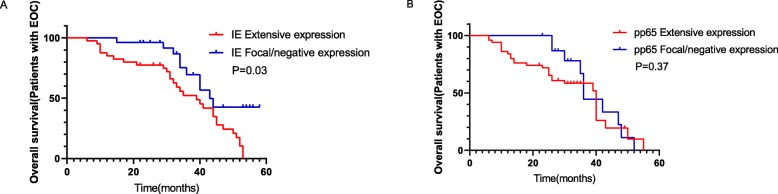
Survival curves of EOC patients with HCMV protein expression. **a** EOC patients who had focal or negative HCMV-IE expression in their tumors had significantly longer median OS than those with extensive HCMV-IE expression (41 vs.39 months, *P* = 0.03). **b** No significant difference in OS was observed in EOC patients with focal/negative or extensive HCMV-pp65 expression in their tumors (42 vs. 40 months, *P* = 0.37)

To confirm the effects of HCMV on EOC clinical outcomes, we analyzed the overall survival by the Kaplan-Meier survival analysis. At study closure, 77% of patients with focal or negative expression of HCMV-IE in their tumors were alive versus 32% of those with extensive expression. The results showed that EOC patients who had focal or negative HCMV-IE expression in their tumors had significantly longer median OS than those with extensive HCMV-IE expression (41 vs.39 months, *P* = 0.03) (Fig. 3a). Similarly, 26% of patients with focal or negative HCMV-pp65 protein expression were alive versus 61% with extensive expression; however, no significant difference in OS was observed(42 vs. 40 months, *P* = 0.37) (Fig. 3b).

To determine whether HCMV-IE expression was an independent risk factor for the OS of EOC patients, we conducted both univariate and multivariate analyses. FIGO stage (*P* = 0.024) and HCMV-IE expression (*P* = 0.032) were prognostic factors for OS in patients with EOC, shown from univariate analysis. Furthermore, the multivariate analysis indicated that HCMV-IE expression (*P* = 0.034) were independent prognostic factors for OS (Table 5).

**Table 5:** Univariate and multivariate analyses of variables for overall survival

Variable	Univariate analysis		Multivariate analysis	
	HR(95%CI)	P	HR(95%CI)	P
Age	0.890(0.724–1.134)	0.332		
Initial CA125	1.124(0.767–1.564)	0.643		
Pathological type	0.893(0.546–1.422)	0.436		
FIGO stage	1.097(1.037–1.946)	0.024	1.154(1.005–1.457)	0.167
HCMV-IE expression	1.008(0.978–1.475)	0.032	1.012(1.003–1.522)	0.034

## Discussion

In our study, we studied HCMV-IE and pp65 proteins. We found that the presence of HCMV-IE and pp65 was detected both in EOC and benign ovarian cystadenoma. However, the positive rates of both proteins in EOC were higher than that in ovarian cystadenoma. This may be due to the fact that the average age of patients with ovarian cystadenoma is lower than that of EOC, while age is one of the risk factors of HCMV infection. Cannon et al. reviewed the literature in order to learn about HCMV transmission and risk factors of infection. They found that HCMV seroprevalence generally increased with age in all 32 studies. In most of the studies that stratified by age, HCMV infection prevalence reached 60% or more in people older than 50 [16]. Moreover, we found that extensive expression of HCMV-IE was correlated with advanced tumor stage. The rate of extensive expression of HCMV-IE in cancer tissue with NACT before surgery was higher than those without NACT. The median OS was shorter among ovarian cancer patients who had extensive expression of HCMV-IE in their tumors than in those with focal or negative expression. And extensive HCMV-IE expression was an independent prognostic factor of OS. These findings suggest that HCMV may have an oncomodulatory effect that contributes to disease progression of EOC patients. To our knowledge, this is the first demonstration of HCMV infection in patients with EOC among Chinese population.

HCMV is a widespread opportunistic pathogen which is estimated to be carried by 40–100% of the world’s population. The infection rate varies according to geographical location, socioeconomic status and age [17]. HCMV can survive in latent form in an immunocompetent host, while it is reactivated during immunosuppression. Several studies have identified high frequency of active HCMV infection in tumor tissues [6–10]. HCMV is considered to be oncomodulatory, although the mechanisms are not clearly understood [18]. The concept of “oncomodulation” suggests that a virus may modulate cellular pathways such as cell proliferation, tumor progression, vascular disease development, and immune evasion [19]. Therefore, HCMV infection may promote malignant transformation by dysregulating the cell cycle and controlling some key physiological processes. Till now, there have been a number of studies suggesting that HCMV proteins such as IE, pp65 and other encoded proteins enable the virus to play an oncomodulatory role [20]. For example, HCMV encodes proteins IE1, IE2, pp71, and pUL97 that can bind or phosphorylate Rb family proteins and inhibit the cell cycle arrest functions of p53. Moreover, HCMV induces a mesenchymal-to-epithelial transition [21]. HCMV-IE often serves as transcription factors that regulate the expression of both viral and host cellular genes, which are crucial to efficient viral replication. IE can also activate production of early and late structural viral proteins, including the viral tegument protein pp65 [22]. HCMV-pp65 is an immunomodulatory protein. It affects expression of HLA-class II and thereby helps the virus to avoid recognition and killing of infected cells by T cells [23]. Thus, HCMV-pp65 expression might worsen patient outcome by mediating an immunosuppressive state in the tumor microenviroment.

In cancers which are not attributable to infectious agents, chronic inflammation may also play a critical role in the transition from a precancerous condition to invasive malignancy [24]. Ovarian cancer is a highly fatal disease and high grade serous ovarian carcinoma (HGSOC) is the most aggressive and common subtype of EOC. Recently the fimbriae of the fallopian tube have been suggested as the precancerous site of HGSOC [25]. Pelvic inflammatory disease (PID), an infection of the female reproductive organs, also results in the possibility of ovarian oncogenesis. Previous studies have implied a potential role of inflammatory factors in the ovarian malignancy process [3]. Inflammation is a key factor of the reactivation of latent HCMV. Active HCMV infection may aggravate the inflammatory microenvironment by increasing production of inflammatory factors such as viral IL-10, tumor necrosis factor-α, transforming growth factor-β and prostaglandins [26]. Baryawno et al. showed a suppressive role of the cyclooxygenase-2 inhibitor in a xenograft model of medulloblastoma, indicating that anti-inflammatory drugs can reduce HCMV replication [27]. Paradowska et al. reported low amounts of viral DNA copies in EOC tissues, suggesting that HCMV exists in ovarian and fallopian tube cells in a latent phase and could be reactivated under the influence of the inflammatory tumor microenvironment [13]. Their study proved the role of HCMV as an oncomodulator rather than involving in direct transformation. Rahbar et al. recently found that prolactin (PRL) and prolactin (PRLR) receptor were induced to high levels in HCMV-infected ovarian cancer cells and PRLR expression was extensively detected in HCMV-infected ovarian tissue specimens [28]. Highly induced PRL and PRLR by HCMV infection may be of relevance for the oncomodulatory role of HCMV in ovarian cancer.

There are also a number of reports concerning HCMV reactivation in patients receiving chemotherapy. In our study, we found that the rate of extensive HCMV-IE protein expression in cancer tissue with NACT before surgery was higher than those without NACT. Chemotherapy might significantly suppress cellular immunity and expose patients to a greater risk of HCMV infection. It is possible that latent HCMV could subsequently be reactivated by the chemotherapy before or the dysregulated inflammatory tumor microenvironment [29].

In this study, multivariate analyses showed extensive HCMV-IE protein expression to be an independent risk factor for the OS of EOC patients. This is in line with Radestad et al. who found that extensive expression of HCMV-pp65 was significantly associated with OS, and median OS was 14 months longer in patients with late-stage serous ovarian carcinoma who had focal expression of HCMV-pp65 in their tumor than in those with extensive expression [15].

Despite surgery and standard therapy, ovarian cancer patients have poor outcomes. Our findings suggest antiviral therapy may have place in future cancer treatment. Preliminary studies in anti-HCMV therapy adding on to standard therapy showed highly improved survival among glioblastoma patients [30]. Future studies are merited to evaluate antiviral treatment in patients with HCMV-positive tumors that have a poor prognosis. The main limitations of the present study were the small sample size and the limited availability of some clinical materials. Further studies with refined design are merited to validate these findings in a larger cohort of patients.

## Conclusion

In the present study, HCMV-IE and pp65 were frequently detected in EOC tissue specimens, and extensive HCMV-IE protein expression was significantly associated with worse outcomes. Evidently HCMV affects the clinical outcomes of EOC, this virus may provide a new therapeutic target in EOC. Our observations suggest that EOC patients who had extensive HCMV-IE expression in their tumors had significantly poor survival rate. Therefore, antiviral therapy may have place in future cancer treatment.

## Data Availability

The data used during the current study is available from the corresponding author on reasonable request.

## Abbreviations

AUC: Area under curve
EOC: Epithelial ovarian cancer
EBV: Epstein-barr virus
FIGO: International Federation of Gynecology and Obstetrics
HCMV: Human cytomegalovirus
IDS: Interval debulking surgery
IL: *Interleukin*
NACT: Neoadjuvant Chemotherapy
OS: Overall survival
PID: Pelvic inflammatory disease
STIC: Serous tubal intraepithelial carcinomas
